# Sparsity-Regularized HMAX for Visual Recognition

**DOI:** 10.1371/journal.pone.0081813

**Published:** 2014-01-02

**Authors:** Xiaolin Hu, Jianwei Zhang, Jianmin Li, Bo Zhang

**Affiliations:** 1 State Key Laboratory of Intelligent Technology and Systems, Tsinghua National Laboratory for Information Science and Technology (TNList), and Department of Computer Science and Technology, Tsinghua University, Beijing, China; 2 Department of Informatics, University of Hamburg, Hamburg, Germany; Georgia State University, United States of America

## Abstract

About ten years ago, HMAX was proposed as a simple and biologically feasible model for object recognition, based on how the visual cortex processes information. However, the model does not encompass sparse firing, which is a hallmark of neurons at all stages of the visual pathway. The current paper presents an improved model, called sparse HMAX, which integrates sparse firing. This model is able to learn higher-level features of objects on unlabeled training images. Unlike most other deep learning models that explicitly address global structure of images in every layer, sparse HMAX addresses local to global structure gradually along the hierarchy by applying patch-based learning to the output of the previous layer. As a consequence, the learning method can be standard sparse coding (SSC) or independent component analysis (ICA), two techniques deeply rooted in neuroscience. What makes SSC and ICA applicable at higher levels is the introduction of linear higher-order statistical regularities by max pooling. After training, high-level units display sparse, invariant selectivity for particular individuals or for image categories like those observed in human inferior temporal cortex (ITC) and medial temporal lobe (MTL). Finally, on an image classification benchmark, sparse HMAX outperforms the original HMAX by a large margin, suggesting its great potential for computer vision.

## Introduction

The primate brain processes visual information in a parallel and hierarchical way. Neurons at different stages of the ventral recognition pathway have different response properties. For example, many retina and LGN neurons are responsive to center-surround patterns, primary visual area (V1) neurons are responsive to bars at particular orientations, V2 neurons are responsive to corners [1], V4 neurons are responsive to aggregates of boundary fragments [2], and inferior temporal cortex (ITC) neurons are responsive to complex patterns such as faces [3].

Motivated by these findings, some hierarchical models have been proposed to mimic the visual recognition process in the brain. One of the earliest representatives is the Neocognitron [4], in which feature complexity and translation invariance are alternatingly increased in different layers. In other words, different computational mechanisms are used to attain the twin goals of invariance and specificity. This strategy has been used in later models, including HMAX [5], which introduces an operation, max pooling, to achieve both scale and translation invariance. It consists of two S layers, two C layers and a view-tuned units layer as an extension of Hubel and Wiesels simple-to-complex cell hierarchy [6]. The S layers perform template matching, that is, higher-level units only fire if their afferents show a particular activation pattern. The C layers perform max pooling, that is, higher-level units are assigned the maximum responses of lower-level units. The higher C layer units and top view-tuned units are able to produce some properties of neurons in the V4 and IT areas of monkeys, respectively [5], [7]. A psychophysical study showed that HMAX accurately predicted human performance on a rapid masked animal versus non-animal categorization task, which suggests that the model may provide a satisfactory description of information processing in the ventral stream of the visual cortex [8].

Despite its success in reproducing some physiological and psychological results, the learning strategy of HMAX is somehow naive. In fact, the low-level features (receptive fields of S1 units) are handcrafted instead of learned. The mid-level features (receptive fields of S2 units) are random patches on the previous layer. An improved version of HMAX has been presented with several important modifications [9], but the learning method still lacks the ability to extract higher-level features.

Sparse coding is an unsupervised learning technique for learning receptive fields of V1 simple cells [10], [11]. It is based on the observation that V1 cells are silent most of the time, firing only occasionally (sparse firing). This model can reproduce the Gabor-like receptive fields of V1 simple cells. Physiological studies have shown that sparse firing is a hallmark of neurons at almost all stages of the ventral pathway, not only in V1. For instance, macaque IT cells fired sparsely in response to video images [12]. A recent study showed that sparse coding better accounted for the properties of receptive fields of macaque V4 cells [13]. This is also true for neurons in the human medial temporal lobe (MTL), which display strong selectivity for only a few stimuli (e.g., familiar individuals or landmark buildings), regardless of their poses and views [14]. All of these results imply that sparse firing plays a significant role in developing an internal representation of the external world. It was thus hypothesized that sparse coding could be used in HMAX to learn different levels of features. This is possible, as we will show that the max pooling operation in the model introduces linear higher-order statistical regularities, which sparse coding can process.

A previous study [15] attempted to combine HMAX and sparse coding to explain the emergence of sparse invariant representations of objects in the human MTL [14], but sparse coding was only applied to the output of the HMAX. Moreover, sparse invariant representations were only probed indirectly by classification accuracy. In this study, we applied sparse coding on each S layer of HMAX to explicitly show that some mid-level and high-level features can emerge by direct visualization. In addition, when applied on mixed categories of images without labels, the proposed model could develop robust internal representations for both coarse (e.g., human faces versus animals) and fine (e.g., faces of different individuals) categorization, which is in agreement with observations in human MTL data [14].

## Methods

### HMAX and sparse coding

**Figure 1:**
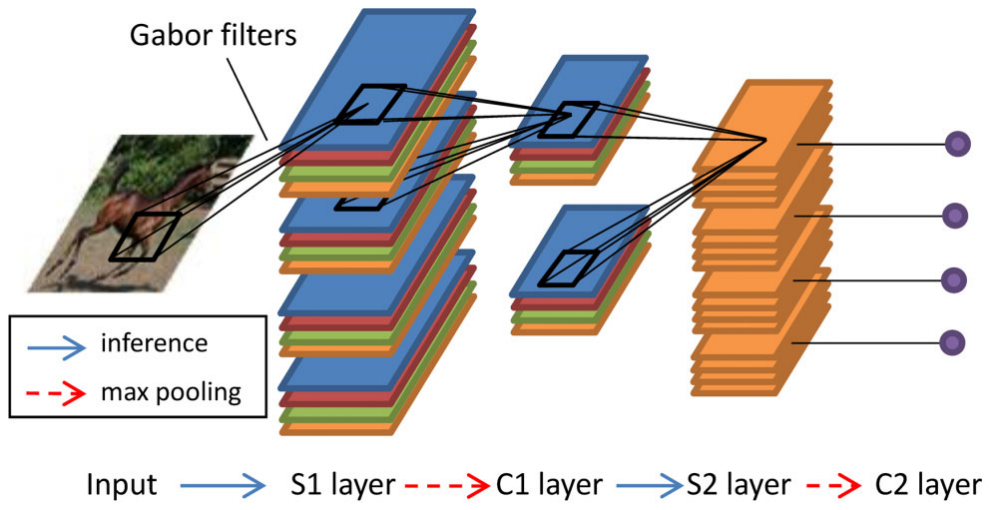
Illustration of HMAX. Inference is realized by template matching. Different colors in S1 and C1 layers correspond to four different orientations of Gabor filters.

Figure 1 shows a typical set-up of the HMAX model [5], [16], [17], which consists of four layers: S1, C1, S2 and C2. A view-tuned units layer can be added after the C2 layer, but is not shown here. A set of handcrafted Gabor filters is convolved with the input image, which results in a set of S1 maps. The S1 maps are grouped in bands according to the filter sizes and positions. Max pooling is applied to the S1 maps with filters within the same band, which results in a set of C1 maps. In the training phase, a set of patches is randomly extracted from the C1 maps as prototypes or bases. All patches on C1 maps are compared with these bases, and the S2 maps are calculated based on the differences; smaller distances yield higher responses. Again, max pooling is applied to S2 maps over all positions and scales to obtain shift- and scale-invariant C2 features.

Note that the Gabor filters used in producing S1 maps are not arbitrary. They are excellent descriptive models for the receptive fields of V1 simple cells [18]. Computational neuroscience suggests that such receptive fields can emerge as a result of sparse coding [11], which essentially extracts dependencies from the visual input that are higher-order than the dependencies between pairs of pixels.

Given 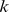 image patches 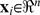, sparse coding seeks a set of bases 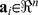 such that 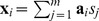 where 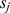 stands for coefficients that are assumed to be sparse, i.e., only a few of them are active (clearly nonzero). In the matrix form, the equation becomes 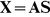
(1)where each column of 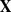 is a patch 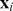, each column of 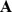 is a basis 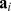 and each column of 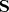 is a vector 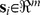 consisting of coefficients of the 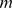 bases for reconstructing 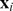. A popular formulation is 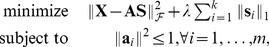
(2)where 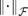 denotes the Frobenius norm and 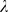 is a positive constant. Inference of latent variable 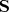 with learned bases 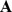 entails solving an unconstrained 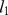 -norm minimization problem.

If 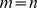 and 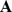 is assumed invertible, then independent component analysis (ICA) [19] can be used to solve (1) and the inference becomes simple: 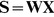
(3)where 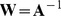. The maximum likelihood formulation of ICA [20] is: 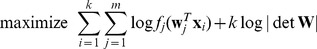
(4)subject to the constraint that the rows of 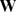 are orthonormal, where 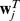 denotes the 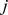-th row of 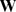, 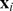 denotes the 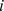-th column of 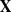 and 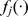 is a sparse probability distribution function.

Throughout the paper *sparse coding models* refer to both model (2) and model (4), as ICA is closely related to sparse coding [19]. To avoid confusion, the model (2) is called *standard sparse coding* or SSC. In addition, columns of 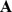 and rows of 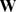 are called *bases* and *filters*, respectively. The bases and filters learned by SSC and ICA are like the receptive fields of V1 simple cells in the cortex.

Sparse coding models are inspired by the observations that neurons in the sensory areas of the cortex remain silent most of the time, firing only occasionally. Experimental data has suggested that sparse firing is a property of neurons throughout the visual hierarchy [12], [14], [21], [22], but HMAX only utilizes this property of V1 neurons. It is natural to ask whether sparse coding can be integrated into higher layers of HMAX (e.g., the S2 layer), and whether it can be used to learn properties of neurons at higher levels of the visual hierarchy (e.g., ITC and MTL). In fact, the S2 layer of the HMAX uses randomly-selected C1 patches as bases, which are unlikely to have a direct correspondence with receptive fields of any neuron. We proposed to replace this simple learning method with sparse coding and extend this strategy to even higher layers. With this modification, learning and inference are consistent across different layers.

Note that both SSC and ICA are linear models, which can at most extract linear statistical regularities from input. Natural images, however, contain nonlinear statistical regularities [19]. In fact, the variances of the linear output are correlated [23], and this finding has motivated many studies to extract nonlinear statistical regularities from images [24]–[26]. Next, we demonstrate that the nonlinear operation used in HMAX, max pooling, enables linear sparse coding models to extract nonlinear statistical regularities from images.

### Emergence of linear statistical regularities after spatial max pooling

In the original HMAX, max pooling is applied over both positions and scales. Since our aim was to construct a model without handcrafting any features, we planned to learn the S1 filters or bases by ICA or SSC. However, it is difficult to estimate the sizes of the learned filters or bases. A simple strategy to construct filters or bases with different sizes is to resize the learned filters or bases and introduce scale pooling in the next step. However, that strategy is not biological feasible. Fortunately, spatial pooling alone can achieve very good results, and only spatial max pooling is considered in this paper. It will be shown that this operation introduces prominent correlations among the uncorrelated output at the same and different locations, which stimulates further exploration of higher-order statistical regularities in images.

**Figure 2:**
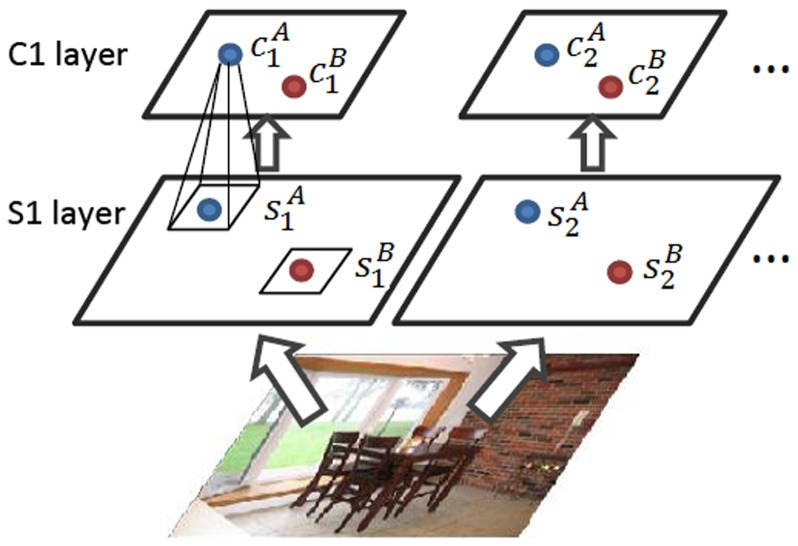
Illustration of the first two layers of HMAX. The subscripts denote filter labels and the superscripts denote positions. Max pooling is only applied over positions.

We learned 64 10-by-10 filters by using ICA on 62 natural scene images from Kyoto dataset (available at http://www.cnbc.cmu.edu/cplab/data_kyoto.html; preprocessed to gray scale) with PCA dimension reduction and whitening [19]. In line with HMAX, each filter was then convolved with the input image to obtain a feature map (S1 layer), which was then down-sampled by max pooling over non-overlapping patches (C1 layer), as shown in Figure 2. Correlation coefficients on the S1 and C1 layers were calculated between responses of:

- different filters at the same location, e.g., 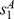 versus 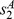 and 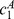 versus 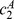 in Figure 2,
- the same filter at different locations, e.g., 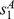 versus 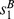 and 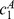 versus 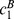 in Figure 2,
- different filters at different locations, e.g., 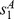 versus 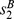 and 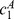 versus 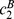 in Figure 2,

On either S1 layer or C1 layer there were 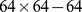 coefficients in the first and third cases (off diagonal entries of a 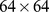 correlation matrix) and 64 coefficients in the second case (diagonal entries of a 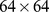 correlation matrix). A total of 100 images were randomly selected from ImageNet (available at http://www.image-net.org/) and their S1 maps were calculated using the 64 filters in the PCA whitened space. C1 maps were obtained by non-overlapping max pooling with pooling ratio 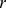: an 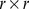 patch on an S1 map was reduced to a single point on the corresponding C1 map, and the value of that point was the maximum value within the 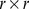 patch. To calculate the first quantity on the S1 layer, 100,000 random locations were used (1,000 locations on each image). To calculate the other two quantities on the S1 layer, another 100,000 locations were used, which were obtained by adding distance 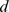 to both vertical and horizontal coordinates of the previously selected 100,000 locations (the first 100,000 locations were selected such that the new locations would not be outside the S1 maps). In the same way, the three quantities were calculated on the C1 layer. To make the distance consistent on the S1 and C1 layers, the distance between different locations on the C1 maps was set to 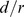.

**Figure 3:**
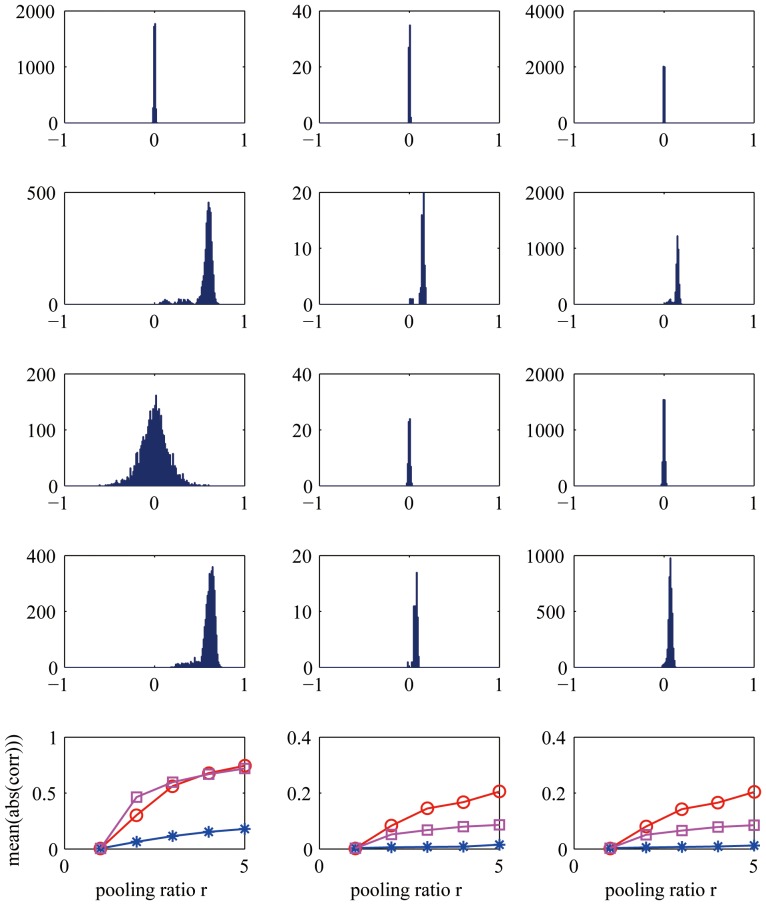
Statistics of correlation coefficients. First column: correlations between responses of different filters at the same location. Second column: correlations between responses of the same filter at different locations. Third column: correlations between responses of different filters at different locations. The distance 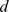 between locations is 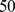 pixels on the original image space. First row: results on the S1 layer in Figure 2. Second to fourth rows: results on the C1 layer in Figure 2 with max pooling, average pooling and square pooling, respectively, where the pooling ratio 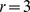. Fifth row: mean of the absolute values of correlation coefficients with respect to the pooling ratio 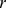, where the open circles, asterisks and squares denote max pooling, average pooling and square pooling, respectively.

The first and second rows in Figure 3 show the histograms of the correlation coefficients on the S1 and C1 layers (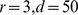), respectively. Clearly, correlations are not present on the S1 layer but appear after spatial max pooling. In addition, the last row shows that correlations are stronger with larger pooling ratios. These observations suggest that the linear higher-order interactions may not exist on the S1 layer, but may exist on the C1 layer. The first hypothesis was validated [27] in a previous study in which a two-layer patch-based sparse coding model without any pooling method produced nothing else than Gabor-like functions. The second hypothesis was validated in the current study (see Results).

The above analysis shows that besides local invariance, max pooling produces second-order linear interactions among the filters, regardless of whether they are at the same or different locations. Note that this is not a unique property of max pooling because other kinds of nonlinear transformations may also have this property. Figure 3 shows the case of square pooling (i.e., square root of the sum of the squares within the 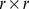 patch on the S1 map). However, average pooling (i.e., average within the 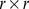 patches on the S1 map) can only produce interactions among different filters at the same location; correlation coefficients are close to zero for the same filter at different locations and for different filters at different locations (Figure 3).

### Sparse HMAX

**Figure 4:**
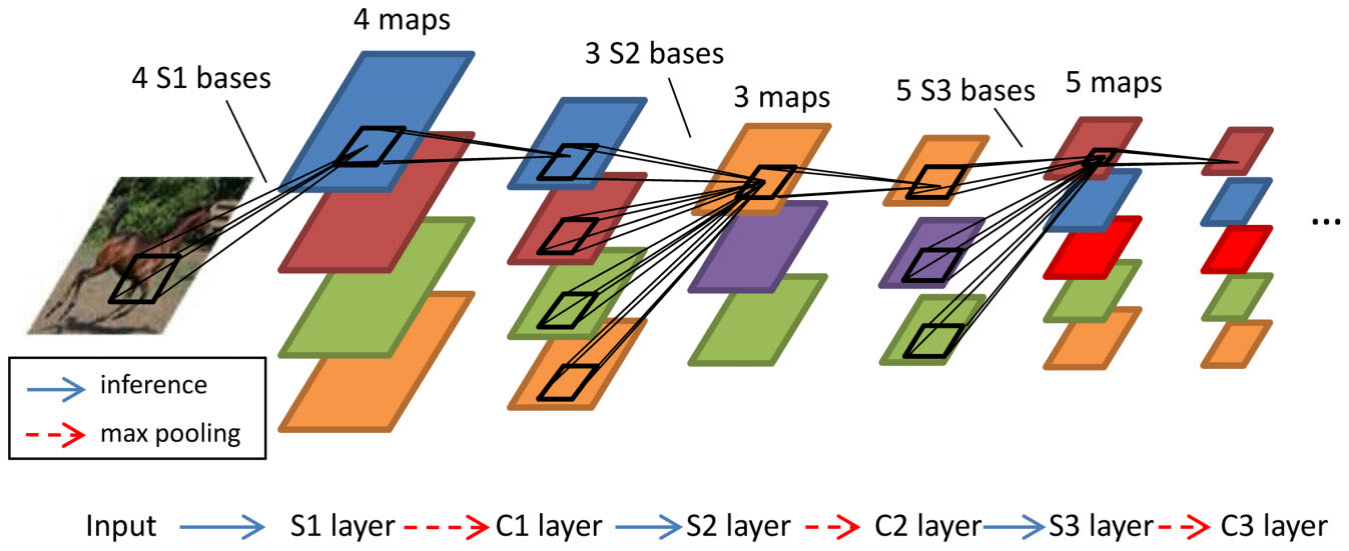
Illustration of sparse HMAX with six layers.

Motivated by the observations of linear dependencies introduced by max pooling, we proposed to learn linear filters or bases by ICA or SSC on each S layer of HMAX. A certain number of bases (called “S bases”) were learned and used to calculate the S layer feature maps. Each C layer consisted of feature maps created by spatially max pooling over the preceding S maps. Figure 4 illustrates three S layers and three C layers, as well as four S1 bases, three S2 bases and two S3 bases. SSC and ICA were used to learn the S bases. SSC can learn overcomplete bases, which is often advantageous for image classification because it produces a large dictionary size. ICA cannot learn overcomplete bases, but is efficient in inferring feature maps because a set of filters can be obtained and convolved with the input to produce the feature maps directly, as in the original HMAX; see (3).

SSC and ICA were applied to small patches sampled from random locations of C maps. Sampling a patch on a C layer means that the same size of patch is sampled on each map at the same location. This implies that the bases learned at C*_k_* layer have dimensions 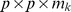, where 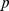 denotes the side length of the patch on each C*_k_* map (without loss of generality patches are assumed to be square) and 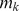 denotes the number of C*_k_* maps.

In our model, the dimensionality of a basis is often higher than the number of bases. For example, a typical S2 basis has dimensions 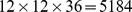, while there are only 100 S2 bases. This is not a problem for SSC because it does not impose a constraint on the two numbers. However, ICA requires that the two numbers are equal. This is achieved by PCA dimension reduction (concurrent with PCA whitening). Since there is a large amount of redundancy in the sampled patches, which is partly introduced by weight sharing, the remaining dimensions may contain enough information for further analysis.

For image classification, the final C maps are used to represent images. In the original HMAX, each S2 random basis leads to a single C2 feature for an image because max pooling is applied over all positions. However, spatial pyramid max pooling has been demonstrated to be more effective [28], and that approach was adopted here. A typical setting for the grid resolutions is 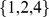, such that each S basis in the final S layer of the sparse HMAX leads to 21 features. In addition, as in previous methods [28], max pooling was applied over the absolute values of the responses within each grid. Note that unlike the S2 codes produced by the original HMAX, the S codes inferred by sparse coding can be either positive or negative.

### Visualization of higher-level features

Similar to [29], the bases of higher-level units were visualized by linearly combining the bases of the units on previous layers. Note that in projecting a basis of a higher-level unit to the input space, the patch size should be enlarged to counterbalance shrinkage due to max pooling. Although it is sufficient to assign a positive value to any unit within a pooling patch and zero values to other units, we found that the visualization effect is better when all units within a pooling patch are assigned the same activation value.

### Comparison with the original HMAX

The major difference between the models is that in the sparse HMAX S2 bases are learned by sparse coding, and therefore the S2 codes are calculated by sparse coding. In contrast, in the original HMAX [16], [17], S2 bases are random patches extracted from C1 maps, and the S2 codes are calculated based on the distance between C1 patches and bases.

Other differences in the sparse HMAX include:

- S1 filters or bases are learned instead of handcrafted;
- S1 and S2 filters or bases have a single size instead of multiple sizes, and as a consequence there is no max pooling over different sizes;
- higher layers than C2 are allowed; and
- a spatial pyramid max pooling [28] is used in the final C layer for image classification instead of max pooling over all positions, which corresponds to a spatial pyramid with the coarsest resolution.

But these differences are not essential and can be easily eliminated. For example, S1 bases can be resized to multiple scales, and S2 bases can be learned in different scales.

### Baseline models

It will be shown that the proposed model enhances the ability of HMAX. However, as discussed above, the two models differ in many aspects, and it is unclear which changes account for the enhancement. Some baseline models with alternative modules are needed to address this issue. We were particularly interested in the contribution of sparse coding, which has two parts, namely bases learning and codes inference. Bases learning can be replaced with a simple strategy used in the original HMAX, that is, randomly extracting patches on the previous S layer. Similarly, codes inference can be replaced with the distance-based approach used in the original HMAX. In this way, some baseline models can be obtained and compared.

In addition, the sparse coding models (2) and (4) can be replaced with the learning rule based on L2 regularization: 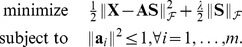
(5)


Inference of latent variable 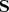 entails solving an unconstrained 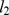-norm minimization problem, which has a closed-form solution. The learning algorithm for the bases 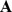 is the same as in [30]. Similar to sparse coding, this model can be used for bases learning, codes inference, or both.

### Stimuli

The stimuli used in this study were all images which were from the following four datasets.

*Kyoto dataset*: This dataset is available at http://www.cnbc.cmu.edu/cplab/data_kyoto.html, which consists of 62 natural scene images of size 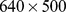 or 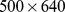.

*Caltech-101*: This dataset is available at http://www.vision.caltech.edu/Image_Datasets/Caltech101/, which contains 9144 images from 102 categories (e.g., animals, human faces, flowers). The number of images per category varies from 31 to 800. Most images are medium resolution (about 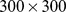 pixels) and well aligned with some variability.

*ImageNet*: This dataset is available at http://www.image-net.org/, which contains a large number of images organized in a hierarchy. Each node of the hierarchy is depicted by hundreds and thousands of images.

*Labeled Faces in the Wild (LFW)*: This dataset is available at http://vis-www.cs.umass.edu/lfw/, which contains more than 13,000 250-by-250 unaligned faces collected from the web. Each face has been labeled with the name of the person pictured.

Caltech-101 was used in Experiments 2 and 4. In Experiment 2, the images were resized such that the shorter side length was 120 pixels while maintaining the aspect ratio. In Experiment 4, they were resized such that the longer side length did not exceed 300 pixels while maintaining the aspect ratio. ImageNet and LFW were used in Experiment 3, where a subset of images were randomly selected from the two datasets, which were resized such that the shorter side length was 150 pixels while maintaining the aspect ratio. All of the images used in the experiments were preprocessed to gray scale.

### Experiments

In Experiments 1 to 3, a five-layer sparse HMAX was trained on different datasets with 36 S1 bases of dimensions 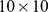, 100 S2 bases of dimensions 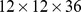 and some S3 bases of dimensions 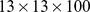. The number of S3 bases varied in Experiments 2 and 3 depending on the task difficulty. The max pooling ratios on S1 maps and S2 maps were 3 and 2, respectively. The bases were learned by ICA with 100,000 patches randomly extracted from images or C patches.

In Experiment 1, the model was trained on all of the 62 images in the Kyoto dataset. In Experiment 2, first, the model was trained on the categories of Caltech-101 separately, and up to 100 images from the same category were used. Second, the model was trained and tested on two distinct sets of images. The training set consisted of 50 images for 10 individuals from the faces-easy category (5 images per individual), 61 images from the car-side category, 32 images from the elephant category and 40 image from the ibis category. The testing set consisted of 100 images from the faces-easy category (10 images per individual), 62 images from the car-side category, 32 images from the elephant category and 40 image from the ibis category. The 10 individuals in the training set and test set were the same. In Experiment 3, the model was trained on 150 LFW images and 1,350 ImageNet images, then tested on 5,000 LFW images and 5,000 ImageNet images. Human faces were excluded manually from the ImageNet images.

In Experiment 4, two architectures were used for performing object classification on Caltech-101 dataset. First, a four-layer architecture was trained. The parameters were as follows: 8 S1 bases of size 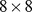 learned by ICA on the Kyoto images; 1024 S2 bases of size 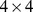 learned by SSC on the Caltech-101 images; pooling ratio 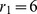. To learn the S2 bases, 200,000 random patches were used with 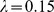 in (2). In the C2 layer, a three-level spatial pyramid at grid resolutions 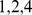 was used on the absolute values of S2 responses. Max pooling was applied to each grid to produce features. Therefore each feature vector had 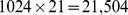 dimensions.

Second, a six-layer architecture was trained with the same S1 and C1 layers as above. Other parameters were as follows: 256 S2 bases of size 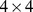 and 1024 S3 bases of size 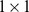 learned by SSC; pooling ratios 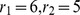; and a spatial pyramid at grid resolutions 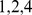 on the C3 layer. Different from previous experiments, to keep enough information on the C2 layer, max pooling on C2 maps was applied on overlapping patches with a step size of 2. The sparsity parameter was 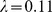 and 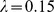 in (2) for learning the S2 bases and S3 bases, respectively. In both cases, 200,000 random patches were used. Note that the first three layers (i.e., S1, C1 and S2) of this architecture were the same as the first three layers of Architecture I, except that only 256 S2 bases were used. To save computational cost, C2, S3 and C3 layers were not stacked directly on top of S2 layer of Architecture I (learning a 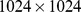 bases matrix is expensive).

To reduce the illumination change effect, inspired by the SIFT features [31], in both architectures, the inferred S responses (except S1 responses) were first normalized to unit length, then thresholded to have no values larger than 0.2 and renormalized to unit length.

Finally, the features extracted by the two models were concatenated together, which resulted in 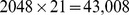 dimensional feature vectors. A multiclass linear SVM was used to perform classification. We followed the common experiment setup for Caltech-101, using 15 and 30 images per category for training and the rest for testing.

The model was implemented in Matlab, and all of the experiments were conducted on a laptop computer (Intel Core i7-3520M CPU 2.90 GHz 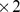 cores, RAM 12.0 GB). The SSC algorithm in [30] and the fastICA algorithm in [20] were used for learning and inference on S layers.

## Results

### Experiment 1: Learning on natural scene images

**Figure 5:**
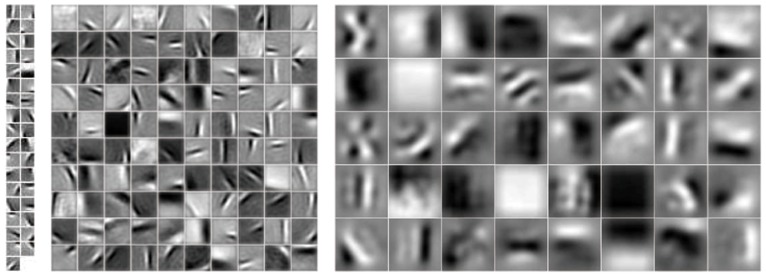
Visualization of S1 bases (left), S2 bases (middle) and S3 bases (right) learned on the Kyoto dataset.

A five-layer model was trained on the 62 Kyoto natural scene images, with 36 S1 bases, 100 S2 bases and 40 S3 bases (see Methods). The learned bases are plotted in Figure 5. Since the pooling ratios 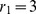 and 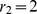, S2 and S3 bases cover roughly three and six times as large an area as the S1 bases, respectively. Note that the bases shown here have been transformed back from the whitened space to the original space. As shown in Figure 5, except a uniform basis, most S1 bases resemble edges, some S2 bases resemble curves, and most S3 bases exhibit complicated patterns. The uniform S1 basis is due to the fact that the mean of sampled patches was not subtracted before PCA whitening. This facilitated bases visualization because some surface information, as well as contour information, could be represented with this uniform basis.

### Experiment 2: Learning higher-level features of objects on aligned images

**Figure 6:**
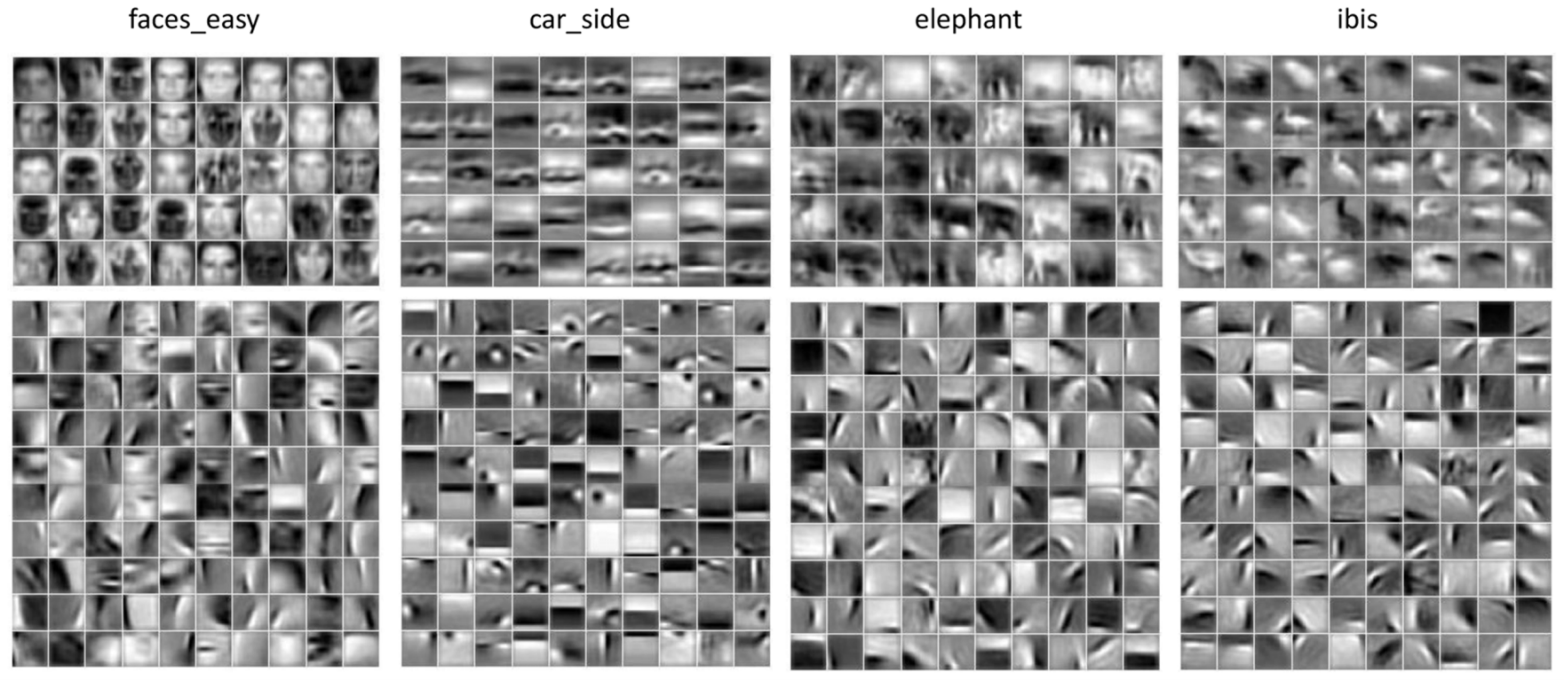
Visualization of S2 bases (bottom) and S3 bases (top) learned on the Caltech-101 dataset. From left to right, the columns display results on images from four categories: faces-easy, car-side, elephant and ibis, respectively.

The same five-layer sparse HMAX was trained on Caltech-101 images, but the S1 bases obtained in Experiment 1 were directly used, since it is well known that the bases learned by ICA are always Gabor-like functions if only it is trained on natural images [19]. For each category of the Caltech-101 dataset, up to 100 images were used for training. Figure 6 displays the respective S2 and S3 bases learned on the categories: faces-easy, car-side, elephant and ibis. It is seen that on some categories (faces-easy, car-side), some S2 bases resemble parts of the objects; on all categories, most S3 bases resemble the whole objects.

**Figure 7:**
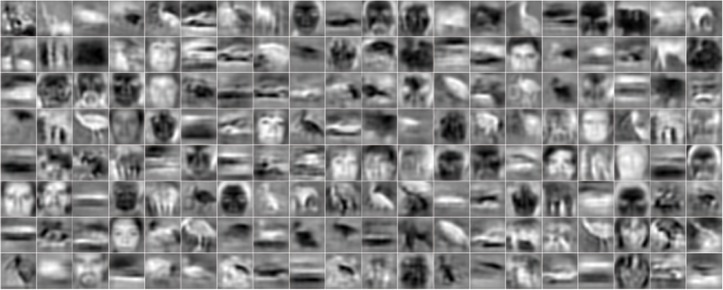
Visualization of S3 bases learned on images from mixed categories of the Caltech-101 dataset: faces-easy, car-side, elephant and ibis.

We then trained the model on images from the four categories together without category labels. In addition, we wanted to quantitatively examine the performance of this unsupervised learning, so we designated both a training set and a testing set, which consisted 183 images and 234 images, respectively (see Methods). The 50 training images from the faces-easy category were derived from 10 individuals (5 images per individual), while the 100 training images from this category were derived from the same 10 individuals (10 images per individual). The model was the same as above except that the number of S3 bases was 160. This is because the task is more difficult than the previous one and more high-level units are needed to represent more concepts. Figure 7 shows the S3 bases learned on the training set, which are object-specific (i.e., they implicitly clustered the images by categories).

To make quantitative analysis possible, we calculated the response of each S3 unit for each input image as the maximum of the absolute values of responses across all locations. A threshold was used for each unit to perform binary classification for a set of inputs. By varying the threshold, a receiver operating characteristic (ROC) curve was obtained and the area under the curve (AUC) was used to characterize the ability of the unit for a particular binary classification task (e.g., ibis versus non-ibis). A unit responding randomly to different classes will have an ROC curve close to the diagonal, while a unit responding selectively to a class will have a curve far from the diagonal, with an AUC close to 1.

**Figure 8:**
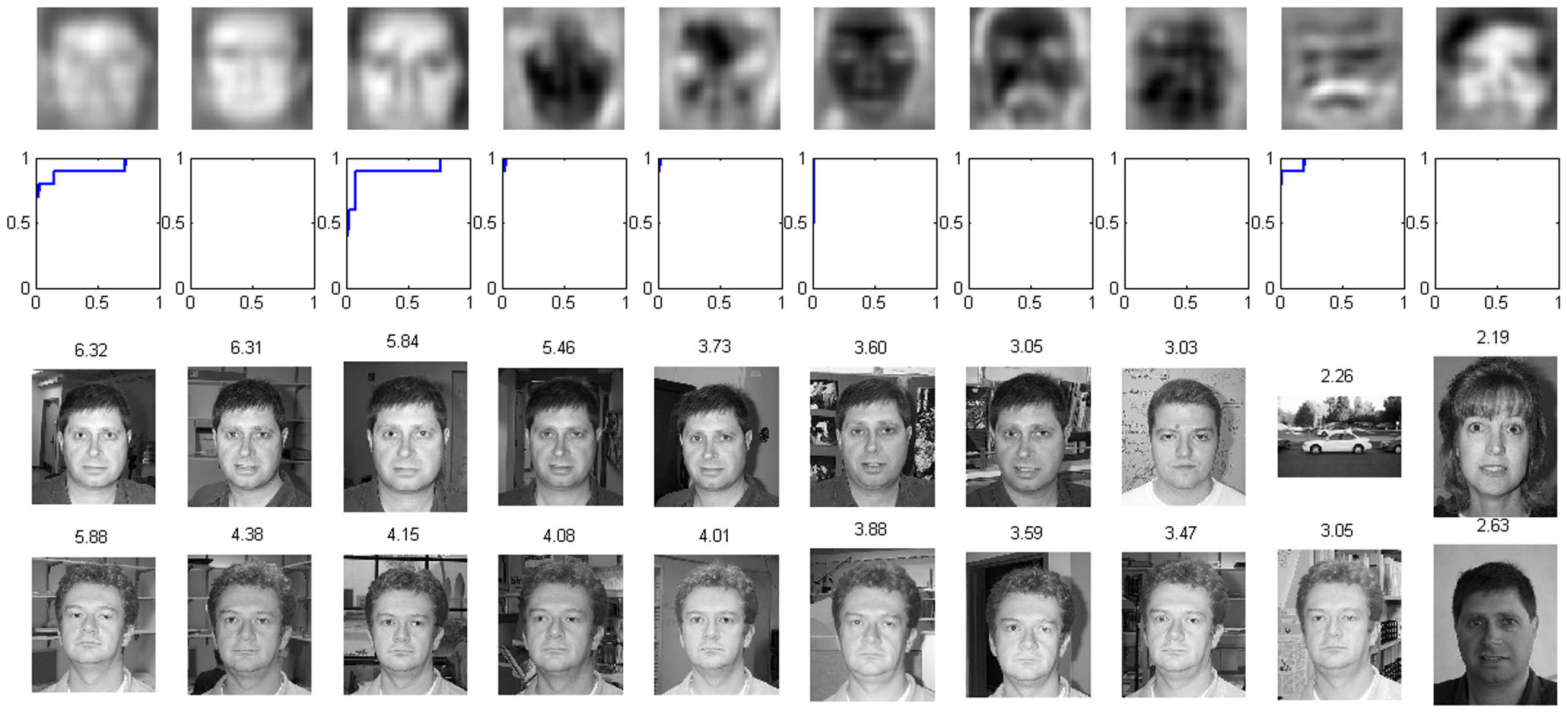
Representation for different individuals. First row: most selective units to ten individuals. Second row: ROC of these units for identifying the corresponding individuals. Horizontal axis: false positive rate. Vertical axis: true positive rate. Third and fourth rows: images that induced highest responses to the first and second units shown in the first row, respectively. The number above each image is the response value of the corresponding unit.

We investigated whether the model had developed invariant representation for particular individuals in the faces-easy category. On the testing set, for each individual, an S3 unit with the highest AUC was defined as the most selective unit for the particular individual. In Figure 8 the top row shows the most selective units for the 10 individuals, and the second row shows their corresponding ROCs. The ROCs were far from the diagonal, indicating excellent representations of the units for different individuals. The third and fourth rows of Figure 8 show the 10 images that induced the highest responses to the first and second S3 units (shown in the top row), respectively. These data suggest that the two neurons encoded two individuals to some extent. Notice that to obtain invariant selectivity to individuals a previous model [15] was trained on face images only, while in our study, the model was trained on not only face images but also other images.

To further explore the properties of the learned S3 units, a multi-category classification was performed similar to the method in [15]. There were 13 categories: 10 categories for individual people and additional categories for car-side, elephant and ibis. First, each unit was assigned a category label according to its maximum AUC over all categories on the training set. Second, for each test image, the predicted label was set to the category label of the unit with the highest response. By comparing the predicted labels and true labels, we obtained an overall accuracy of 84.91%, which is much higher than the chance level of 7.69%.

**Figure 9:**
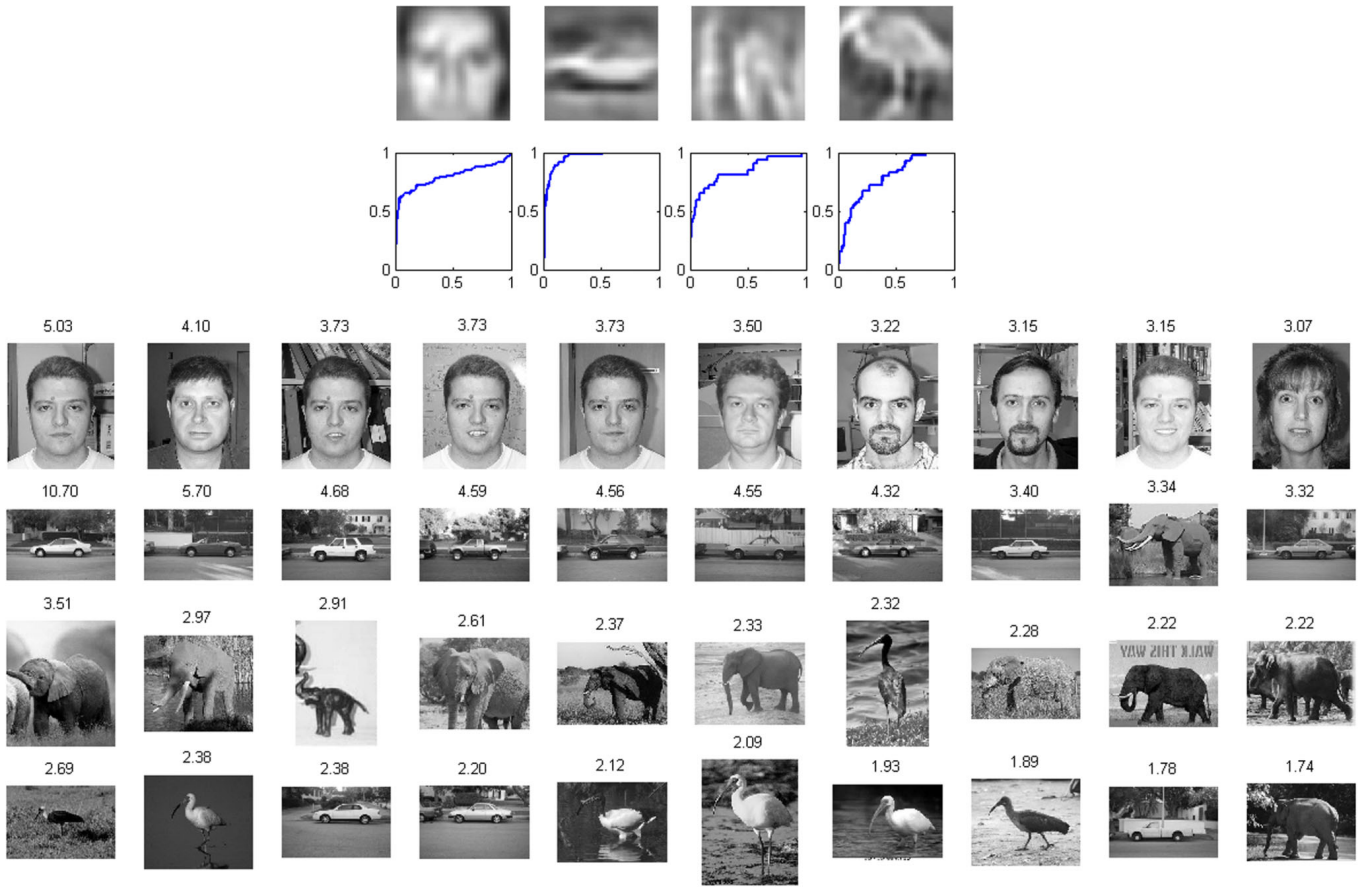
Representation for general categories. First row: most selective units to the four categories. Second row: ROC of these units for identifying the corresponding categories. Horizontal axis: false positive rate. Vertical axis: true positive rate. Third to sixth rows: images that induced highest responses to the four units shown in the first row, respectively. The number above each image is the response value of the corresponding unit.

To determine whether the model had developed invariant representation for more general categories, all face images were labeled as one category, and car-side, elephant and ibis comprised additional categories. Figure 9 shows the most selective unit for each category according to the AUC criterion. The four units have some ability to represent these four categories.

### Experiment 3: Learning higher-level features of objects on unaligned images

The Caltech-101 images are aligned. It is more desirable to learn higher-level features from unaligned images. To investigate the formation of face-specific neurons in the human ITC, we attempted learning of “face neurons” on unaligned and unlabeled images. A total of 150 LFW images and 1,350 ImageNet images were used for training. And a total of 5,000 LFW images and 5,000 ImageNet images were used for testing, where the LFW images served as positive samples and ImageNet images served as negative samples.

Since there was more variability in the training set than in Experiment 2, more high-level units are needed. We used 400 S3 bases. The experiment took about 2 hours on the 1,500 training images. Each learned S3 unit was then associated with a threshold. A test image was classified as positive (face detected) if the maximum response to that image exceeded the threshold; otherwise it was classified as negative. For each S3 unit, we tested 20 equally-spaced thresholds between the minimum and maximum activation values to the training images. The optimal threshold, based on classification accuracy, was then selected and used to classify the 10,000 test images.

**Figure 10:**
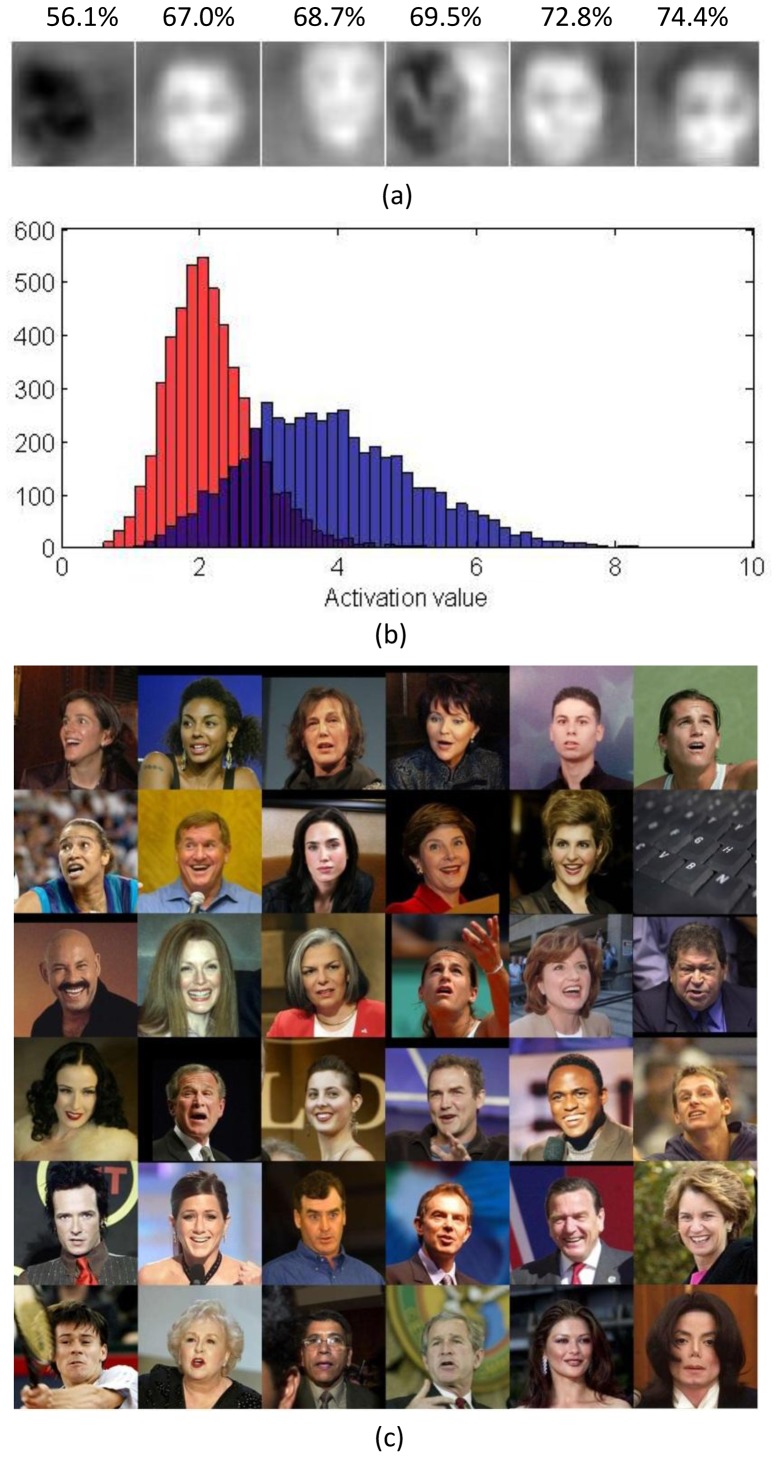
Training and testing on a mixture of LFW data and ImageNet data. (a) Bases of six face-sensitive units with their test accuracies indicated above. (b) The histogram of the activation values of the best unit (the rightmost in (a)) for 5,000 positive samples (blue) and 5,000 negative samples (red). (c) 36 images that elicited greatest activations for the best unit.

Only a few of the 400 units were selective to faces (i.e., the testing accuracy above the chance level of 50%). Figure 10(a) shows six units with their testing accuracy indicated on top of them. Figure 10(b) shows the histograms of maximum activation values of the best unit to the positive and negative samples, respectively, in the test dataset. Face images and distractors elicited two distinct activation patterns for the unit. Figure 10 (c) shows 36 images that elicited maximum activations of the best unit; most of them contain faces.

**Figure 11:**
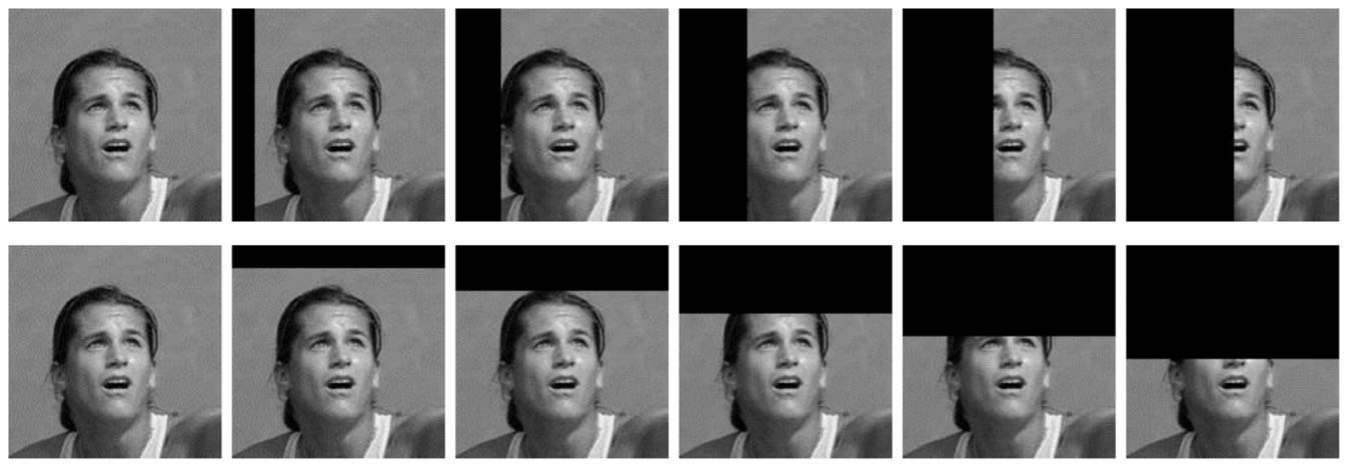
A sample sequence of horizontal occlusions (top) and vertical occlusions (bottom). All of the occlusion portions shown here correspond to the activation values above the threshold of the second best unit (see the last row of Figure 12).

**Figure 12:**
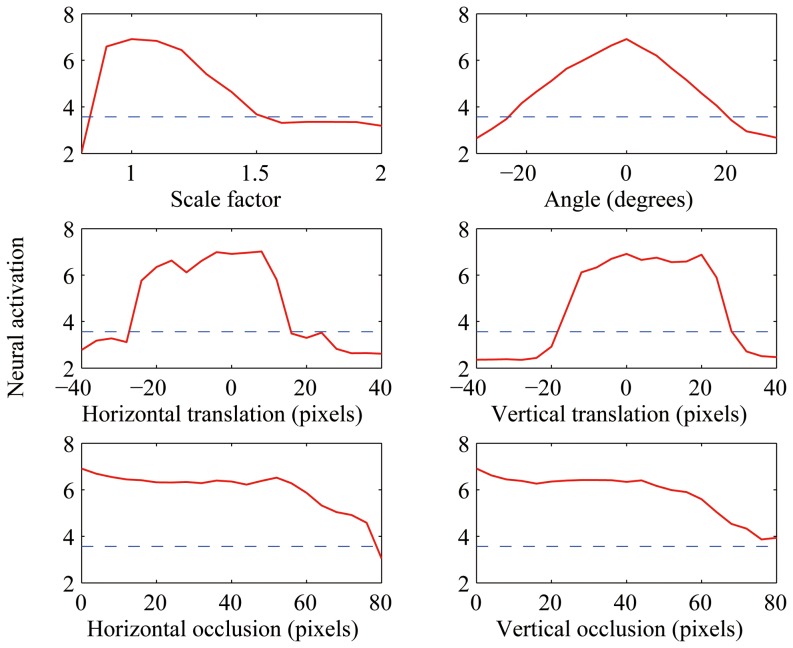
Average activation value of the second best unit on distorted images. Dashed line indicates the threshold.

Finally, similar to [32] we tested the invariance properties of these “face neurons”. We applied some standard image distortions including scaling, rotation and translation to ten randomly selected face images. We also applied occlusion; samples of this distortion are shown in Figure 11. All units were resistant to these distortions within a range, suggesting that they encoded some higher-level features. Average responses of a sample unit (fifth unit from Figure 10(a)) over the distorted images are plotted in Figure 12.

In the training set, about 10% of the images contained faces. We found that both this proportion and the number of S3 units influenced the final results. Specifically, with 10% face images in the training set, the model with 160 S3 units failed to produce any face-specific units. However, with 30% face images in the training set, the model with 160 S3 units produced at least one face-specific unit. These results suggest that more units are required for emergence of higher-level features of those objects that appear with lower probabilities in the training set.

### Experiment 4: Object classification

The sparse HMAX was used to classify objects from the Caltech-101 dataset. Two architectures were trained, called Architecture I and Architecture II, with four and six layers, respectively (see Methods). A multiclass linear SVM was used to perform classification. Table 1 details the results, which were averaged over 10 random splits of train/test samples. The table also summarizes the results of some recent models learning from the pixel level. To the best of our knowledge, the results reported in [33] and [34] represent the state-of-the-art. Although a higher accuracy was reported in [35], a saliency map was used, which is quite different from the models listed in Table 1. Architecture I outperformed previous HMAX models by a large margin, and the combination of Architectures I and II produced even higher accuracies.

**Table 1:** Classification accuracy in percent on the Caltech-101 dataset.

Training Size	15	30
Architecture I	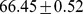	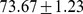
Architecture II	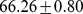	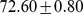
Architectures I+II	**68.98±0.64**	**76.13±0.85**
Random bases + distance	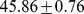	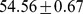
Random bases + SSC	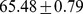	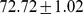
HMAX [17]	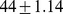	-
Mutch and Lowe [9]	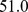	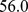
Lee et al. [29]	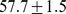	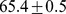
Kavukcuoglu et al. [38]	-	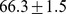
Zeiler et al. [39]	-	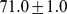
Yu et al.[33]	-	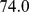
Zou et al. [34]	-	74.6

The results are shown as 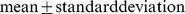.

Architecture I has the same number of layers as the original HMAX [16], [17]. To evaluate the contribution of the sparse coding introduced in the S2 layer, we replaced the SSC in Architecture I with the learning method used in the original HMAX. Specifically, 1024 C1 patches of size 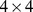 were randomly extracted as bases and the S2 responses were calculated based on the distances from these bases (shorter distance indicates higher responses). Other settings were the same as in Architecture I. As shown in Table 1, the performance of the model is not much better than the performance of the original HMAX (see “Random bases + distance” in the table). Interestingly, using the same set of random bases but with SSC for inference, the results are very similar to the performance of Architecture I (see “Random bases + SSC” in the table). These data suggest that the performance enhancement of the sparse HMAX is mainly due to sparse coding especially the inference algorithm.

**Figure 13:**
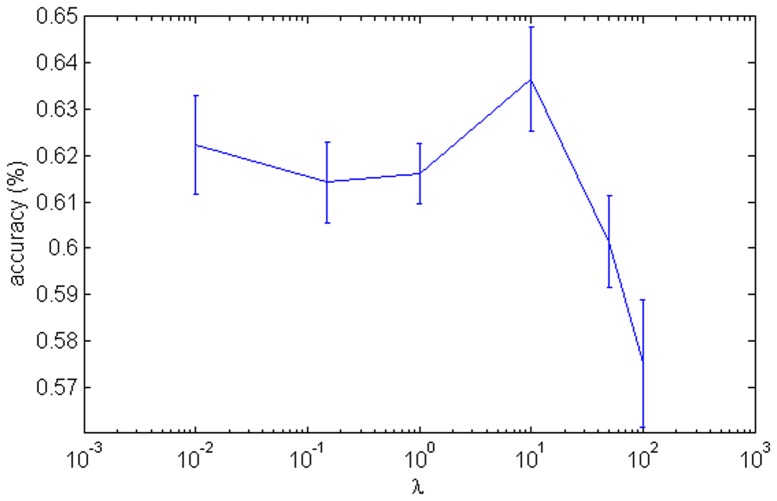
Classification accuracy of the L2-regularized HMAX with respect to different values of the regularization parameter 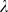
 **on the Caltech-101 dataset.** The curve shows the average results over ten random splits of train/test samples and the error bars show the standard deviations. The x-axis is in the log scale.

To further clarify the contribution of sparse coding, we replaced the SSC in Architecture I with the L2-regularized model (see Methods). The resulting model was tested with different regularization coefficients 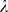. Figure 13 shows the results with 30 training samples per category. At 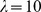, the best accuracy of 63.66% was achieved, which was 10% lower than the accuracy of Architecture I.

## Discussion

The major contribution of this paper is the integration of sparse coding techniques into HMAX, a well-known cortex-inspired visual recognition model, to create a simple yet powerful variant model called *sparse HMAX*. With SSC or ICA, more and more abstract concepts can be learned along the hierarchy. We have demonstrated that sparse HMAX can learn an invariant representation of objects and maintains some selectivity, which is in agreement with observations from human ITC and MTL.

### Nonlinear pooling and sparse coding

A hallmark of HMAX is max pooling, which has an advantage over average pooling [5] because with this operation one can tell if a pattern inducing a particular output is actually present or not, which preserves some pattern specificity. This study revealed another advantage of max pooling over average pooling: it introduces linear higher-order statistical regularities among the filter outputs at different locations, which facilitates learning of higher-level features using sparse coding techniques. Figure 3 suggests that square pooling would also have this property, and this has been verified in some models [24], [32]. Some other nonlinear transformations have been shown to be able to introduce linear higher-order dependencies [36], [37], but they are much more complicated and cannot induce larger receptive fields because they operate on different filters at the same location.

The two closely related techniques, SSC and ICA, are well-accepted as being able to learn the receptive fields of V1 simple cells. They lay a theoretical foundation for sparse firing of neurons in V1 from a computational viewpoint. Sparse firing is also a property of many neurons beyond the V1 stage [12]–[14], [21], [22]. From a metabolic viewpoint, understanding the prevalence of sparse firing across the visual hierarchy is easy. However, its computational implications at higher stages are unclear. This work, together with other recent studies e.g., [29], [32], suggests that theoretical implications of sparse firing of V1 neurons could be extended to higher stages along the visual hierarchy.

The success of HMAX is mainly due to alternating template matching and max pooling, which increase both selectivity and invariance along the hierarchy [5]. Our modification, that is, imposing a sparse firing constraint, takes effect on the template matching step, which essentially amplifies the selectivity of the model. Adding sparsity to the top layer of HMAX can produce some results in agreement with the data recorded from the MTL [15], but our results indicated that integrating this property into every layer of the model is more advantageous for learning higher-level representations of objects.

### Comparison with existing models

The current results show some similarity to those of many other hierarchical visual recognition models (often called deep learning models) that have been proposed in recent years (e.g., [27], [29], [32], [33], [38]–[40]). This is because the sparse HMAX essentially uses similar principles to these former models. One of the major differences is that sparse HMAX uses patch-based sparse coding algorithms, while many other models often integrate convolution into the sparsity-regularized algorithms to address global structure of the image [27], [29], [38], [39] (but see [40]), which has led to complicated algorithms. In other words, sparse HMAX addresses local interactions on the current layer and allows longer range interactions to be handled by subsequent layers containing units with larger receptive fields. This has two nontrivial consequences. First, it makes the sparse HMAX efficient because many efficient and scalable learning algorithms exist [20], [30], [41]. Second, patch-based learning makes it easier to understand information processing in the visual cortex using information theory [19]. For example, the minimum entropy coding and information transmission principles could be studied at these stages with minimum adaptation.

The success of traditional patch-based sparse coding algorithms, together with the simple max pooling, validate the key roles of sparsity regularization and nonlinear pooling employed by previous deep learning models. In this sense, the proposed model, an improved version of HMAX, can be also viewed as an extension of these models.

Another difference lies in the biological feasibility. In fact, prior models contain learning blocks, such as restricted Boltzmann machine (RBM) [42], auto-encoder [43] and agglomerative clustering. Furthermore, these models often entail complicated optimization techniques, such as convolutional sparse coding [39]. These approaches have not been well justified from the viewpoint of neuroscience. In contrast, the two operations in the proposed HMAX, max pooling and patch-based sparse coding, are considered to be biologically-feasible because they have detailed biophysical models [11], [44]–[46].

### Comparison with physiological data

The current results are only in agreement with some general observations from physiological data (e.g., the emergence of sparse and invariant representation of both fine and coarse categories). It remains a challenge to quantitatively test how accurate the model is for the visual cortex. One obstacle is the scarceness of unbiased physiological data in higher stages of the visual pathway. It is difficult to accurately quantify the response properties of higher-level neurons in the brain due to their tolerance to variations. The traditional strategy is to present predefined stimuli to animals and measure neuronal responses in the target area (e.g., [1]). The results obtained are inevitably biased to the stimuli used. Some recent techniques, such as adaptive stimuli design [13], may provide a better solution.

Another way to test the validity of the model is to extensively train animals, as well as the model, on novel stimuli and compare the experimental data with the model output. This strategy would confine the search for optimal stimuli for neurons within a small space. It is predicted that some controlled mid-level and high-level patterns would emerge in both cases.

### Applications to computer vision

As a cortex-inspired model, HMAX was first used to perform image classification in 2005 [16]. But it has lagged behind other computer vision models (e.g., [27], [28], [33], [39]) despite development of some improved versions [9], [17]. Equipped with sparsity regularization, we have shown that HMAX outperforms many state-of-the-art models learning from the pixel level on an object classification benchmark, indicating that it is still a useful model for computer vision.

In [32], a sparse deep autoencoder was trained on 10 million images, taking three days on a cluster with 1,000 machines (16,000 cores). Finally it obtained “face neurons”, “cat neurons” and “body neurons” from images derived from Youtube videos. In [40], a deep learning model based on two classical clustering algorithms and trained on 250 cores was also shown to be able to learn “face neurons”. We have shown that sparse HMAX can also learn “face neurons”, but with much lower computational resources (2 cores). Though the problem is simplified, the result strongly suggests that sparse HMAX may be more efficient for image classification on large scale datasets.

The performance of the model could be improved if biological plausibility is not a concern. For example, different scales of filters can be constructed by resizing the learned bases and max pooling over scales can be integrated into the model. Some scale invariance will result, which is definitely helpful for visual recognition. Another possibility would be to integrate saliency detection techniques as in [35].
